# Free Field Word recognition test in the presence of noise in normal hearing adults^☆^

**DOI:** 10.1016/j.bjorl.2016.08.015

**Published:** 2016-09-27

**Authors:** Gleide Viviani Maciel Almeida, Angela Ribas, Jorge Calleros

**Affiliations:** a Universidade Tuiuti do Paraná (UTP), Programa de Mestrado e Doutorado em Distúrbios da Comunicação, Curitiba, PR, Brazil; b Laboratório de Acústica e Equipamentos Audiológicos, Curitiba, PR, Brazil

**Keywords:** Auditory perception, Hearing tests, Noise, Hearing, Percepção auditiva, Testes auditivos, Ruído, Audição

## Abstract

**Introduction:**

In ideal listening situations, subjects with normal hearing can easily understand speech, as can many subjects who have a hearing loss.

**Objective:**

To present the validation of the Word Recognition Test in a Free Field in the Presence of Noise in normal-hearing adults.

**Methods:**

Sample consisted of 100 healthy adults over 18 years of age with normal hearing. After pure tone audiometry, a speech recognition test was applied in free field condition with monosyllables and disyllables, with standardized material in three listening situations: optimal listening condition (no noise), with a signal to noise ratio of 0 dB and a signal to noise ratio of −10 dB. For these tests, an environment in calibrated free field was arranged where speech was presented to the subject being tested from two speakers located at 45°, and noise from a third speaker, located at 180°.

**Results:**

All participants had speech audiometry results in the free field between 88% and 100% in the three listening situations.

**Conclusion:**

Word Recognition Test in Free Field in the Presence of Noise proved to be easy to be organized and applied. The results of the test validation suggest that individuals with normal hearing should get between 88% and 100% of the stimuli correct. The test can be an important tool in measuring noise interference on the speech perception abilities.

## Resumo

**Introdução:**

Em situações ideais de escuta, indivíduos com audição normal conseguem realizar e reconhecer a fala facilmente. Porém, na presença de ruído competitivo, é comum as pessoas sentirem dificuldades de compreensão, principalmente se tiverem perda auditiva.

**Objetivo:**

Apresentar a validação do teste de reconhecimento de palavras em campo livre na presença do ruído em indivíduos adultos normo-ouvintes.

**Método:**

A amostra foi composta por 100 sujeitos hígidos, com audição normal e maiores de 18 anos. Depois da audiometria tonal foi aplicado teste de reconhecimento da fala, com monossílabos e dissílabos, em campo livre, com material padronizado, em três situações de escuta: condição ótima de audição (sem ruído), com ruído em relação de 0 dB, e com ruído em relação de -10 dB. Para tanto, montou-se um ambiente de teste em campo livre calibrado, onde a fala foi apresentada ao indivíduo em teste com duas caixas acústicas localizadas a 45° e o ruído em uma terceira, localizada a 180°.

**Resultados:**

Todos os participantes tiveram resultados de logoaudiometria em campo livre entre 88 e 100% nas três situações de escuta.

**Conclusão:**

O teste de reconhecimento de palavras em campo livre na presença do ruído mostrou-se de fácil organização e aplicação. Os resultados obtidos na validação do teste sugerem que indivíduos com audição normal devem acertar entre 88 e 100% dos estímulos apresentados. O teste pode configurar um instrumento importante na mensuração da interferência do ruído sobre as habilidades de percepção de fala.

## Introduction

To understand speech satisfactorily, some auditory tasks are necessary, including: attention, analysis, synthesis, memory, among others. Such skills, when combined, promote auditory recognition, which implies deriving meaning from what is heard. Thus, the understanding of speech is a very complex activity that depends directly on the peripheral hearing mechanisms, central auditory processing and cognition.^1^

In ideal listening situations, that is, acoustically comfortable environments, individuals with normal hearing easily can perform the auditory recognition. However, when the environment is degraded, due to the competitive noise or reverberation,^2^ it is common for people to have difficulty understanding.

In individuals with hearing loss and hearing aid users (conventional or implantable) this difficulty is greater.^3^, ^4^

Among other uses, the speech perception test in the presence of noise has been developed and used in audiological diagnosis^5^ to evaluate central auditory processing^1^ and to select and evaluate the performance of hearing aids.^6^ Most available tests use supra-aural headphones or insert earphones^1^; a minority are performed in a free field setting.^7^ Some of these tests require expensive technological apparatus that can make it less available for use in routine speech therapy.

Because of this, a low-cost, easy to install audiology laboratory was designed, consisting of an acoustically treated booth and a free field system attached to three speakers, to perform speech recognition testing in the presence of noise.

The aim of this paper is to present the validation of the Free Field Word Recognition Test in the Presence of Noise in normal-hearing individuals.

## Methods

This is an experimental, self-controlled study, aimed to verify the accuracy of the Free Field Word Recognition Test in the Presence of Noise. The study was approved by the Institutional Ethics Committee under protocol 937 031/15.

One hundred individuals who agreed to participate were randomly selected. All of them were oriented about the study and signed the Informed Consent.

The study included persons 18 years of age or greater on the date of the tests, who had normal hearing and no hearing complaints. We excluded individuals with speech problems.

For data collection a booth, a two-channel audiometer, conventional open field equipment (for speech stimulus output) and auxiliary equipment (for noise emission) were used.

### The equipment

The auxiliary equipment called “third channel” for free field has been developed specifically to control and amplify a third sound source used as “competitive sign” inside the booth. This one has the circuit composed of the following blocks: input preamplifier, calibration circuit with gain adjustment of 0–40 dB, linear output attenuator with 5 dB steps, and full range of 0–100 dB SPL, 50-W class-T digital power amplifier, PIC18F2550 MICROCHIP microcontroller, a 2-lines by 20 characters Display, and a keyboard.

The system operation allows the adjustment of the external sound source, which in this project was a Samsung mobile, with an SG (Sound Generator) application; the application was set to generate (broadband) white noise. The professional can calibrate the correct signal level used by the “calibration mode” of the equipment by viewing the signal on the display, and adjusting it to “0 dB” on the VU. Once adjusted, the signal can be displayed in the selected intensity of the attenuator through the stimulus button that turns the presentation of this noise on or off.

**Figure 1:**
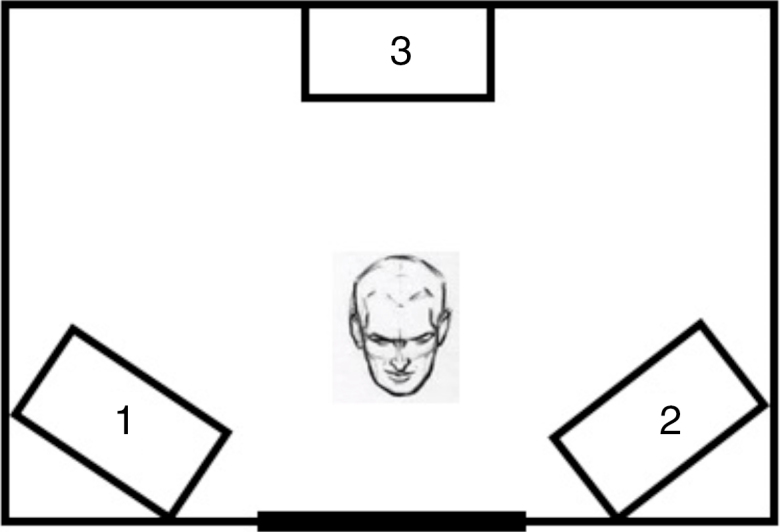
Positioning of speakers inside the booth. 1 – speaker at 45° on the left; 2 – speaker at 45° on the right; 3 – speaker at 180° with noise.

Test environment consisted of two speakers located at 45° (right and left of the evaluated subject) and noise in a third one, located 180° from the subject from where competitive noise is produced (Fig. 1).

The conventional free field used is Oto Sonic CL30-V model, with no series number, calibrated on October 17, 2013, with certificate No. 415-2013F, according to ISO8253-3 and IEC645-2:1993, the standards used for calibration were Larson Davis Sound Pressure Meter, Mod. 824, series No. 824A2867 (Certificate No. 50381/2013), Larson Davis Sound Calibrator, mod. CAL 250, series no. 4128 (Certificate No. 50378/2013), Larson Davis Microphone, Mod. 2575, series no. 1698 (Certificate No. 50379/2013).

The “third channel” equipment was calibrated on July 31, 2014, with certificate no. 425a-2014-F, according to ISO8253-3 and IEC645-2:1993, the standards used for calibration were Bruel & Kjaer Sound Level Meter, Mod. 2250, series No. 3006245 (Certificate No. CBR1400264/2014), Bruel & Kjaer Acoustic Calibrator, mod. 4231, series No. 3007539 (Certificate No. CBR1400268/2014) and Larson Davis Microphone, Mod. 2575, series no. 2119 (Certificate No. 60381/2014).

### Data collection

After completing a form of identification, all research subjects underwent pure tone audiometry to determine the auditory thresholds. Those with hearing within normal limits^8^ were submitted to the speech recognition test, using standardized and recorded material.^9^

The recognition test was applied in three situations:

1. Monosyllabic word lists presented without competitive noise (control);
2. Monosyllabic word lists presented in signal/noise ratio of 0 dB (study);
3. Monosyllabic word lists presented in signal/noise ratio of −10 dB (study).

The word lists were presented at 40 dB NS, or 40 dB above the mean tritone levels previously obtained in pure tone audiometry. In the signal/noise ratio of 0 dB, speech and noise were presented at the same intensity. With a signal/noise ratio of −10 dB, the noise was 10 dB stronger than speech. The speech was presented in the conventional free field systems (speakers at 45°) and the competitive noise was presented at the “third channel” (180°).

Data were recorded in a special record protocol, and were statistically analyzed. We used the chi-square test at the 0.05 significance level.

The following variables were analyzed and compared: test results with no noise (control) with test results with noise at 0 dB and −10 dB ratio (study).

After this, the study subjects were divided into two groups: G1 – people under 40 years of age, and G2 – people with 40.1 years or more, and data were compared to see if the scores varied with increasing age.

## Results

One hundred normal-hearing subjects were evaluated for purposes of this study, 83 being females and 17 males. The minimum age of the sample was 19 years and the maximum 64 years. The mean age was 34.1 years, with a standard deviation of 10.8.

Given the standard deviation found, and to verify if age factor interfered with the results of auditory perception, the sample was divided into two groups, G1 composed of 72 individuals under 40 years and G2 formed by 28 subjects over 40.1 years.

All subjects of the sample demonstrated free field speech recognition scores between 88% and 100% accuracy on the three listening conditions (Table 1).

**Table 1:** Descriptive statistics of speech perception with no noise and with competitive noise.

Conditions	*n*	Mean	Median	Minimum	Maximum	Standard deviation
No noise	100	99.96	100.00	96.00	100.00	0.40
With noise 0 dB	100	98.28	100.00	92.00	100.00	2.22
With noise −10 dB	100	96.04	96.00	92.00	100.00	3.03

Speech perception performance obtained in the Free Field Word Recognition Test in the Presence of Noise was compared between the two groups (Table 2). There was no statistically significant difference.

**Table 2:** Comparison of G1 and G2 means.

Variable	G1 above 40 years			G2 over 40.1 years			*p*
	*n*	Mean	Standard deviation	*n*	Mean	Standard deviation	
Speech with no noise	72	99.9	0.47	28	100.0	0.00	0.5356
Speech with noise 0 dB	72	98.2	2.22	28	98.4	2.27	0.6787
Speech with noise −10 dB	72	96.3	3.10	28	95.4	2.82	0.2106

## Discussion

The ability to understand speech in the presence of competitive noise is the object of the study of audiology,^10^ because it is an important phenomenon that greatly interferes in people's quality of life, especially those who are users of conventional or implantable hearing aids.^11^, ^12^ Associated with this research, the use of standardized (recorded) material has been developed^13^, ^14^ to ensure the reliability of results, a fact that is strictly followed in this study.

When analyzing the mean score of respondents (Table 1) in the Free Field Word Recognition Test in the Presence of Noise proposed here, it was found that there was no significant difference when considering the three variables (listening conditions), with the scores obtained being virtually identical, ranging from 99.96–96.4%. In normal-hearing individuals, between 88% and 100% accuracy is expected in speech recognition tests in optimal listening environment.^9^, ^13^, ^15^

When the respondents were divided into two subgroups (Table 2) for the investigation of the age factor on the test, it was observed that the answers were also similar, with no significant differences between them. The literature^10^, ^11^, ^16^, ^17^ indicated that with age, auditory processing tends to be difficult due to several factors, but this was not the object of this study.

Normally, the broad spectrum noise tends to hinder the task of auditory discrimination because speech consists of sounds of different frequencies that have continuously varying intensities,^18^ and these sound characteristics can be masked by noise, and result in important perceptual confusions.^19^ In difficult listening conditions, cognitive overload can occur, resulting in significant frustration to both the listeners and the speakers, and greater language skills are needed to improve speech perception, that is also associated with the increase of intensity load.^20^

The competing noise proposed in our test was presented at an angle of 180°, in accordance with Brazilian research^21^ on speech recognition thresholds in normal-hearing individuals in the presence of noise that demonstrated that in free field condition, the best thresholds are achieved with incidence angles of 0°–90° and 0°–270°, followed by the condition 0°–180° and, finally, 0°–0°. It is noteworthy that in the daily life environments the noise and speech fall on people from different angles, but a test that reproduces these conditions is not feasible in the speech therapy practice, due to cost and time.

It is noteworthy that, in audiology clinics, it is extremely important to establish reference values obtained in normal-hearing subjects in order that the difficulties encountered by the individual with hearing disorder complaints can be compared. Considering the findings reported in this study, values between 90% and 100% accuracy can be inferred as normal standard of the Free Field Word Recognition Test in the Presence of Noise in subjects with no hearing complaints.

The test proposed here consisted of a low-cost, easy to install system that provided reliable results that were consistent with the literature. It can be a valuable tool in the investigation of auditory processing, selection and indication of hearing aids, as well as in the evaluation of performance of patients using sound amplification and implantable prostheses, and can provide a framework for research and testing of different populations in the future.

## Conclusion

The Free Field Word Recognition Test in the Presence of Noise proved to be easy to organize and apply. The results of the test validation suggest that subjects with normal hearing should get between 90% and 100% of the stimuli presented correct, even in the presence of noise. The test can be an important tool in measuring noise interference on speech perception skills in different populations.

## Conflicts of interest

The authors declare no conflicts of interest.
